# Childhood acute leukemias are frequent in Mexico City: descriptive epidemiology

**DOI:** 10.1186/1471-2407-11-355

**Published:** 2011-08-17

**Authors:** María Luisa Pérez-Saldivar, Arturo Fajardo-Gutiérrez, Roberto Bernáldez-Ríos, Armando Martínez-Avalos, Aurora Medina-Sanson, Laura Espinosa-Hernández, José de Diego Flores-Chapa, Raquel Amador-Sánchez, José Gabriel Peñaloza-González, Francisco Javier Álvarez-Rodríguez, Victoria Bolea-Murga, Janet Flores-Lujano, María del Carmen Rodríguez-Zepeda, Roberto Rivera-Luna, Elisa María Dorantes-Acosta, Elva Jiménez-Hernández, Martha Alvarado-Ibarra, Martha Margarita Velázquez-Aviña, José Refugio Torres-Nava, David Aldebarán Duarte-Rodríguez, Rogelio Paredes-Aguilera, María de los Ángeles del Campo-Martínez, Rocío Cárdenas-Cardos, Paola Hillary Alamilla-Galicia, Vilma Carolina Bekker-Méndez, Manuel Carlos Ortega-Alvarez, Juan Manuel Mejia-Arangure

**Affiliations:** 1 Unidad de Investigación en Epidemiología Clínica, Unidad Médica de Alta Especialidad UMAE Hospital de Pediatría, Centro Médico Nacional (CMN) Siglo XXI, Instituto Mexicano de Seguridad Social (IMSS), México D.F., México; 2 Servicio de Hematología, UMAE Hospital de Pediatría, CMN "Siglo XXI", IMSS, México D.F., México; 3 Servicio de Oncología Pediátrica, Instituto Nacional de Pediatría (INP), Secretaría de Salud (SSa), México D.F., México; 4 Servicio de Onco-Hematología, Hospital Infantil de México Federico Gómez, SSa, México D.F., México; 5 Servicio de Hematología Pediátrica, Hospital General "Gaudencio González Garza", CMN "La Raza", IMSS, México D.F., México; 6 Servicio de Hematología Pediátrica, CMN "20 de Noviembre", Instituto de Seguridad Social al Servicio de los Trabajadores del Estado, México D.F., México; 7 Hospital General Regional "Carlos McGregor Sánchez Navarro", IMSS, México D.F., México; 8 Servicio de Onco-Pediatría, Hospital Juárez de México, SSa, México D.F., México; 9 Servicio de Oncología, Hospital Pediátrico de Moctezuma, Secretaría de Salud del D.F., México D.F., México; 10 Hospital General de México, SSa, México D.F., México; 11 Subdirección de Hemato/Oncología, INP, SSa, México D.F., México; 12 Servicio de Hematología Pediátrica, INP, SSa, México D.F., México; 13 Unidad de Investigación Médica en Inmunología e Infectología, Hospital de Infectología Daniel Méndez Hernández, "La Raza" IMSS, México D.F., México; 14 Coordinación de Salud en el Trabajo, CMN Siglo XXI, IMSS, México D.F., México

## Abstract

**Background:**

Worldwide, acute leukemia is the most common type of childhood cancer. It is particularly common in the Hispanic populations residing in the United States, Costa Rica, and Mexico City. The objective of this study was to determine the incidence of acute leukemia in children who were diagnosed and treated in public hospitals in Mexico City.

**Methods:**

Included in this study were those children, under 15 years of age and residents of Mexico City, who were diagnosed in 2006 and 2007 with leukemia, as determined by using the International Classification of Childhood Cancer. The average annual incidence rates (AAIR), and the standardized average annual incidence rates (SAAIR) per million children were calculated. We calculated crude, age- and sex-specific incidence rates and adjusted for age by the direct method with the world population as standard. We determined if there were a correlation between the incidence of acute leukemias in the various boroughs of Mexico City and either the number of agricultural hectares, the average number of persons per household, or the municipal human development index for Mexico (used as a reference of socio-economic level).

**Results:**

Although a total of 610 new cases of leukemia were registered during 2006-2007, only 228 fit the criteria for inclusion in this study. The overall SAAIR was 57.6 per million children (95% CI, 46.9-68.3); acute lymphoblastic leukemia (ALL) was the most frequent type of leukemia, constituting 85.1% of the cases (SAAIR: 49.5 per million), followed by acute myeloblastic leukemia at 12.3% (SAAIR: 6.9 per million), and chronic myeloid leukemia at 1.7% (SAAIR: 0.9 per million). The 1-4 years age group had the highest SAAIR for ALL (77.7 per million). For cases of ALL, 73.2% had precursor B-cell immunophenotype (SAAIR: 35.8 per million) and 12.4% had T-cell immunophenotype (SAAIR 6.3 per million). The peak ages for ALL were 2-6 years and 8-10 years. More than half the children (58.8%) were classified as high risk. There was a positive correlation between the average number of persons per household and the incidence of the pre-B immunophenotype (Pearson's r, 0.789; P = 0.02).

**Conclusions:**

The frequency of ALL in Mexico City is among the highest in the world, similar to those found for Hispanics in the United States and in Costa Rica.

## Background

Acute leukemias (AL), especially acute lymphoblastic leukemias (ALL), have been reported with a very elevated incidence within the Hispanic pediatric population in the United States (USA) [1-3]. For children under the age of 15 years, the incidences of ALL worldwide varies between 20-35 cases per million [4], whereas the incidence of ALL for Costa Rica and in Mexico City (also known as the *Distrito Federal*) and for the Hispanic populations that live in the USA are greater than 40 cases per million [1-6].

The incidence rates of leukemias that have been reported for Mexico City correspond fundamentally to that portion of the population, i.e., private-sector employees and their families, entitled to the services provided by the *Instituto Mexicano del Seguro Social *(IMSS), which comprises approximately 40% of the entire population [6,7]. In those studies, the overall incidence of leukemia was found to be between 55.4 and 58.4 per million [6,7]. The incidences for various leukemias are the following: ALL, between 43.2 and 44.9 per million; acute myeloid leukemias (AML), between 9.8 and 10.6 per million [6,7]; chronic myeloid leukemias (CML), 2.5 per million; and unspecified leukemias (UL), 0.5 per million [6]. The proportion of T-lineage ALL has been reported at 23.6% [8], a frequency relatively higher than that reported for the White population of the United States of America (USA) or for Asiatic populations [8]. For our population, the proportion of children with ALL at high risk vs. standard risk is 1:1, a proportion higher than that reported by other institutions in the USA [9].

Nevertheless, in Mexico City, there exist other population groups that have not been represented in these studies, because they do not fall under the aegis of IMSS. For government workers (which include such groups as public school teachers, civil service workers, and public servants) and their families, social security and medical care are provided by a separate agency, the *Instituto de Seguridad Social al Servicio de los Trabajadores del Estado *(ISSSTE) [10,11]. The unemployed in Mexico City receive medical attention at the hospitals of the *Secretaría de Salud *(SSa) and at the hospitals under the aegis of the *Distrito Federal*. Therefore, included in this study were all the public hospitals (i.e., dependent on the government) that provide medical care for children with leukemia in Mexico City. It has been shown that these public hospitals treat 97.5% of all the cases of leukemia occurring in Mexico City [12]. At present, there is no population-based registry of childhood leukemias which encompasses the population either of Mexico City or of the whole of Mexico, nor is there one concerning childhood cancers in general [7]. For this reason, the participation of all the hematological and oncological medical personnel of the public hospitals in Mexico City was necessary in order to be able to identify actively all the new cases of AL diagnosed within the study period.

In Mexico, few studies have dealt with the question of whether socio-economic level has an influence on the incidence of leukemias [13]. From the foregoing, the objectives of the present study were 1) to determine the incidence of acute leukemias in children from Mexico City; 2) to determine the frequency of the T phenotype and the ratio of high risk to standard risk for the children with leukemia who reside in Mexico City; and 3) to determine the correlation between the incidence of ALL, T-cell ALL, Pre-B ALL, and AML with municipal human development index (MDHI), number of agricultural hectares, and average number of persons per household.

## Methods

### Design

Population-based, descriptive study.

### Population studied

For a case to be included in this study, the patient had to meet the following criteria:

• Be a child under 15 years of age;

• Reside in Mexico City;

• Be newly diagnosed with leukemia during the years 2006-2007, with the diagnosis confirmed by histopathology;

• Be diagnosed and treated in a public hospital in the *Distrito Federal*. (See next section, *Hospitals*).

All such cases from 2006 to 2007 were analyzed.

### Hospitals

Although medical attention for children with AL is provided by different health institutions, both public and private, the public sector has been estimated to treat 97.5% of the cases of AL that occur in Mexico City [10]. Of the 13 hospitals that are equipped to treat children with AL in Mexico City, four were not included in this study because it had been determined in previous studies that these four hospitals (two hospitals of *Petróleos Mexicanos*, a *Hospital Militar de México*, and the Hospital Regional No. 25 of IMSS) had not treated cases of children from Mexico City. The nine hospitals that were included represent various governmental agencies: IMSS, SSa, ISSSTE, and the *Secretaría de Salud del Distrito Federal *(SSDF). These hospitals, in descending order of the proportion of cases each contributed to this study, were the following: *Instituto Nacional de Pediatría *(SSa), 27.6%; *Hospital Infantil de México "Federico Gómez" *(SSa), 20.2%; *Hospital de Pediatría del Centro Médico Nacional "Siglo XXI" *(IMSS), 19.3%; *Hospital General "Gaudencio González Garza" of the Centro Médico Nacional "La Raza" *(IMSS), 18.0%; *Centro Médico Nacional "20 de Noviembre" *(ISSSTE), 6.6%; *Hospital Regional "Carlos McGregor Sánchez Navarro" *(IMSS), 3.5%; *Hospital Juárez de México *(SSa), 2.6%; *Hospital Pediátrico de Moctezuma *(SSDF), 1.8%; and *Hospital General de México *(SSa), 0.4%.

### Sources of patient data

In each participating hospital, we had a trained nurse or medical assistant for identifying cases of suspected acute leukemia. For each such case, after having signed a consent form, the parents were interviewed to determine demographic variables, and the patient's record was reviewed to obtain the clinical variables and the diagnosis. To ensure the quality of our data, the information was collected independently of the only existing Mexican Registry of Childhood Cancer (MRCC), which is maintained by IMSS and which contains data only for those served by IMSS [7]. The concordance between the data in our register for patients served by IMSS and the data in the MRCC was 100%.

### Diagnosis

Once diagnosed with presumed leukemia, a child was referred to one of the hospitals where trained staff (hematologists and onco-hematologists) did a comprehensive follow-up of the case to either confirm or discard the diagnosis of leukemia. Bone marrow smear was used to confirm each diagnosis; histochemical tests (myeloperoxidase, Sudan black B reaction, esterases, periodic acid Schiff (PAS) reaction, and acid phosphatase) were performed to differentiate the types of leukemia.

Morphological classification was used to divide the leukemias into five groups, according to the International Classification of Childhood Cancer (International Classification of Disease for Oncology) [14]. Only four of the five types were found in this study: a) ALL (9820-9827, 9850); b) AML (9840, 9841, 9861, 9864, 9866, 9867, 9891, 9894, 9910); c) CML (9863, 9868); and d) UL (9800-9804). The files were reviewed to corroborate that there were no duplicate records or inconsistent data. A database was generated to record age, sex, residency, year of diagnosis, and clinical manifestations of the patients.

Immunophenotyping was performed by flow cytometry. Cases of ALL were classified according to one of the following immunophenotypes: precursor-B cell, mature-B cell, T lineage, or "not otherwise specified", if the registered information did not allow proper classification. For the cases of ALL, the immunophenotypes registered were the following: for precursor B-cell ALL, CD19+, HLADR+, cyCD22+, CD10+ or CD10, TdT+, CD20+, cyCD79a+, and CD34+; for T lineage ALL, CD19, CD22, CD79a, CD7+, CD5+, cyCD3+, clgm, and sIg; and for mature B cells, immunoglobulin (kappa or lambda light chain as surface markers).

### Risk Criteria

Only two risk categories were used for this study: 1) children, aged 1-9 years, with a white blood cell count less than 50,000/μL were classified as being at standard risk; 2) children either who were in the 10-14 age group or who were younger and had a white blood cell count greater than 50,000/μL were designated as being at high risk [15].

### Populations

Because the size of the base population, estimated from the data for Mexico City from the *Instituto Nacional de Estadística, Geografía e Informática *(INEGI) [16], was known, it was feasible to obtain the incidence rate for the population under 15 years of age (Additional file 1 Table S1). The denominator was calculated by using data from the intermediate census of 2005. In 2005, the *Distrito Federal *had a population of 8.72 million inhabitants, 2.04 million of whom were children under the age of 15 years [16]. With no census having been carried out in 2006 or 2007, there is no official count for those years; therefore, as estimation for those two years, the 2005 value for the population under 15 years of age was multiplied by two. (The population of Mexico City has remained quite stable from 1990 to date, with changes of only 0.04% per year [16].)

### Analysis

AAIR were calculated in total, by kind of leukemia, by age group (< 1 year, 1-4 years, 5-9 years, or 10-14 years), and by sex. The standardized average annual incidence rates (SAAIR) were standardized by age by using the direct method with the world standard population [17], reported per million.

To determine whether the SAAIR of leukemias varied by some characteristic in the boroughs of Mexico City, the correlation, as calculated by using Pearson's **r**, between the incidence of ALL and that of AML and the MHDI [18] for Mexico was determined. The value of P was reported, with P = 0.05 used as the cutoff value for statistical significance. The MHDI is a measurement of how well cities and their subdivisions are doing. The values of this index range between 0 and 1, with values closer to 1 signifying a greater degree of well-being.

Three indicators are used to construct this index:

• Long and healthy life (measured by life expectancy at birth);

• Educational level (measured by the adult literacy rate and the combined raw rates of matriculation in primary, secondary, and higher education, as well as the length of compulsory education); and

• Standard of living (measured by the gross domestic product (GDP) per capita purchasing power parity (PPP) in dollars).

Information concerning this index was available only for the year 2005 [16]. Each borough of Mexico City was analyzed both according to the hectares used for agriculture contained within its boundaries and according to the average number of persons per household, information that also was available only for the year 2005, [16] (data not shown).

This study was approved by the Ethics Board of the National Commission of Scientific Investigation (Registry No. 2008-785-063).

## Results

During the period of the study, the number of new cases of childhood leukemias diagnosed in public hospitals in Mexico City was 303 in 2006 and 307 in 2007. Of these 610, only 228 children were residents of the *Distrito Federal *(37.4%). Of the 228 patients, 194 (85.1%) had ALL; 28 (12.3%), AML; four (1.7%), CML; and two (0.9%) UL (Table 1).

**Table 1:** Number of cases, average incidence rates, and standardized average annual incidence rates for children from Mexico City by kind of leukemia, sex, and age group (in years), 2006-2007

				Age	Group		(Years)					
**Childhood**	**Sex**		**< 1 y**		**1-4 y**		**5-9 y**		**10-14y**		**Total**	SAAIR*
**Leukemia**		**n**	**AAIR**	**n**	**AAIR**	**n**	**AAIR**	**n**	**AAIR**	**n**	**AAIR**	
					
ALL	M	4	31.9	48	87.1	26	38.1	22	30.8	100	48.2	
	F	2	16.5	36	67.8	36	54.6	20	28.7	94	46.8	
	Total	6	24.3	84	77.7	62	46.2	42	29.8	194	47.5	49.5
AML	M	1	8.0	3	5.4	3	4.4	6	8.4	13	6.3	
	F	0	0	5	9.4	4	6.1	6	8.6	15	7.5	
	Total	1	4.1	8	7.4	7	5.2	12	8.5	28	6.9	6.8
CML	M	0	0	1	1.8	0	0	1	1.4	2	1.0	
	F	0	0	0	0	0	0	2	2.9	2	1.0	
	Total	0	0	1	0.9	0	0	3	2.1	4	1.0	0.9
UL	M	0	0	0	0	0	0	1	1.4	1	0.5	
	F	0	0	1	1.9	0	0	0	0	1	0.5	
	Total	0	0	1	0.9	0	0	1	0.7	2	0.5	0.5

AAIR: average annual incidence rate; SAAIR: standardized AAIR; ALL: acute lymphoid leukemia; AML: acute myeloid leukemia; CML: chronic myeloid leukemia; UL: unspecified leukemia; M: male; F: female; *Expressed per million children.

### General incidence of leukemias in children in the Distrito Federal

The overall SAAIR was 57.6 per million (95% CI, 46.9-68.3), with the SAAIR for ALL being 49.5 per million; for AML, 6.8 per million; and for CML, 0.9 per million. The male:female ratio was 1.0 for leukemias in general, 1.1 for ALL, 0.9 for AML, and 1.0 for CML.

### Incidence of leukemia by age group

The AAIR for ALL was highest (77.7 per million) for the 1-4 year age group. In this age group, the AAIR for ALL for males was higher than that for females (87.1 and 67.8 per million, respectively), whereas for the 5-9 year age group, the AAIR for ALL was higher for females (Table 1).

### Incidence of leukemia by immunophenotype

The immunophenotypes were determined for 96.4% of the ALL cases: 73.2% of the total ALL cases were classified as precursor B-cell; 12.4% as T cell; 8.2% as B cell; and 2.1% as dual phenotype, with 0.5% as undeterminable. The SAAIR of the pre-B ALL was 35.8 per million and that of the T-cell ALL was 6.3 per million (Table 2).

**Table 2:** Age-group average incidence rates of two immunophenotypes in acute lymphoid leukemia in children from Mexico City (2006-2007)

				Age	Group	(years)					
			
Immuno-phenotype		< 1 y		1-4 y		5-9 y		10-14 y		Total	
in ALL	n	AAIR*	n	AAIR*	n	AAIR*	n	AAIR*	n	AAIR*	SAAIR*
T-Cell	0	0	13	12.0	8	6.0	3	2.1	24	5.9	6.3
Pre B	3	12.2	58	53.6	46	34.2	35	24.8	142	34.8	35.8

AAIR: average annual incidence rate; SAAIR: standardized AAIR; ALL: acute lymphoid leukemia; Pre B: Precursor B cell. *Expressed per million children.

### Ratio of high risk to standard risk for ALL

Of the ALL patients, 58.8% were classified as being high risk; when only those cases of ALL having precursor B- cell immunophenotype were considered, 53.5% were high risk.

### Morphological subtypes of AML

Of the cases of AML, the most frequent classifications were M2 with seven cases; M4, with six; and M1 and M5, with five cases each. M3 represented only 10.7% of the cases.

### Age peak for leukemias

**Figure 1:**
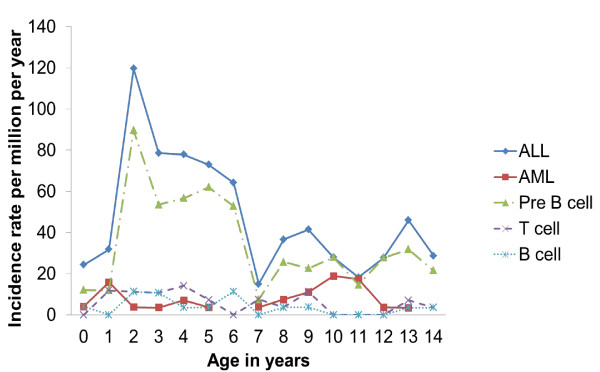
**Comparison of age-specific incidence rates of childhood leukemia in Mexico City (2006-2007)**. The age-specific incidence rates of acute lymphoid leukemia (ALL), of acute myeloid leukemia (AML), and of ALL immunophenotypes (precursor B-cell, T cell, and B cell) for Mexico City children (2006-2007) were compared.

As determined from the graph (Figure 1) the peak age for AL was found to be between 2-6 years of age for ALL, with another noteworthy peak at 8-10 years of age. These age peaks corresponded to those for precursor B-cell ALL. For T-cell ALL, a small peak was seen between 1-4 years of age. The AML showed a peak at one year and between 10-11 years (Figure 1).

### Incidence of leukemias by borough and correlation with MDHI, number of cultivated hectares, and average number of persons per household

The SAAIRs for the boroughs of Mexico City ranged from 23.0 to 87.7 per million, with Cuauhtémoc, a relatively affluent borough, having the highest SAAIR (Table 3).

**Table 3:** Average annual age-standardized incidence by kind of leukemia and immunophenotype in boroughs of Mexico City (2006-2007)

Boroughs of Mexico City		Leukemia	type			Immunophenotype			
	
		ALL		AML		T-Cell		Pre-B Cell	
	
	MHDI†	(n)	SAAIR*	(n)	SAAIR*	(n)	SAAIR*	(n)	SAAIR*
Alvaro Obregón	0.8719	8	23.0	2	5.0	1	2.5	6	17.1
Azcapotzalco	0.8915	5	29.4	3	16.5	3	17.4	2	12.0
Benito Juarez	0.9509	5	48.3	0	0	0	0	4	38.9
Coyoacan	0.9169	14	59.6	2	8.7	1	4.8	12	50.0
Cuajimalpa de Morelos	0.8994	3	30.1	0	0	0	0	3	30.1
Cuauhtémoc	0.8921	16	87.7	1	5.0	2	11.6	8	44.0
Gustavo A. Madero	0.8700	34	62.7	2	3.0	6	11.0	24	43.8
Iztacalco	0.8765	8	48.6	0	0	1	6.7	5	28.4
Iztapalapa	0.8463	45	47.0	10	10.9	4	4.6	33	33.7
La Magdalena Contreras	0.8558	6	54.3	0	0	0	0	6	54.3
Miguel Hidalgo	0.9188	6	43.7	2	14.1	1	6.6	5	37.1
Milpa Alta	0.7983	4	61.5	1	16.5	0	0	4	61.5
Tláhuac	0.8473	10	54.8	0	0	2	11.8	6	31.8
Tlalpan	0.8791	9	31.2	2	5.9	0	0	9	31.2
Venustiano Carranza	0.8740	12	63.7	3	14.7	1	6.2	8	41.4
Xochimilco	0.8481	9	48.4	0	0	2	9.4	7	39.1
		**194**	**49.47**	**28**	**6.76**	**24**	**6.26**	**142**	**35.80**

SAAIR: standardized average annual incidence rate; ALL: acute lymphoid leukemia; AML: acute myeloid leukemia; CML: chronic myeloid leukemia; UL: unspecified leukemia; Pre B: Precursor B cell. *Expressed per million children. ^†^MHDI: *Municipal Human Development Index for Mexico 2005 *[18].

**Figure 2:**
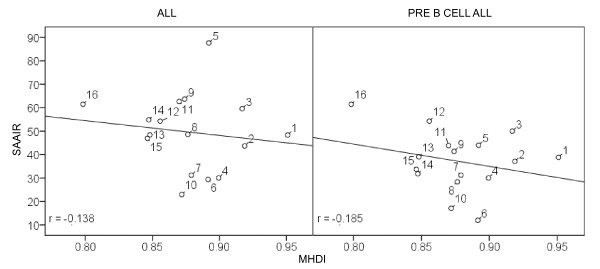
**Correlations between municipal human development indices of boroughs of Mexico City and incidences of ALL and of precursor B-cell immunophenotype**. Panel A: Incidence of ALL; Panel B: Incidence of precursor B-cell immunophenotype. SAAIR: standardized average annual incidence rate by million of children below under 15 years of age; MHDI: municipal human development index; Boroughs of Mexico City: 1) Álvaro Obregón; 2) Azcapotzalco; 3) Benito Juárez; 4) Coyoacán; 5) Cuajimalpa de Morelos; 6) Cuauhtémoc; 7) Gustavo A. Madero; 8) Iztacalco; 9) Iztapalapa; 10) La Magdalena Contreras; 11) Miguel Hidalgo; 12) Milpa Alta; 13) Tláhuac; and 14) Tlalpan; 15) Venustiano Carranza; and 16) Xochimilco. Source: *Municipal Human Development Index (MHDI) for Mexico 2005 *[18]. *Expressed per million children.

The MHDI of the boroughs of Mexico City (Figure 2A, B) showed a negative correlation both with the incidence of ALL (Pearson's r, -0.138; P = 0.30) and with the incidence of precursor B-cell ALL (Pearson's r, -0.185; P = 0.49); in both instances, there was little precision in the estimation. No correlation was found between the number of cultivated hectares and the incidence of either AML, ALL, or precursor B-cell immunophenotype. However, there was a statistically significant, positive correlation between the average number of persons per household and the incidence of the pre-B immunophenotype (Pearson's r, 0.789, P = 0.02).

## Discussion

The incidence rates of AL differ in the various countries of the world, depending in great measure on the socio-economic level of the populations [19]: the higher the socio-economic level, the higher the incidence of AL [20]. However, the incidences among Hispanics are distinct, as Hispanic populations have the highest incidence rates of AL. The population of Mexico City exemplifies this situation [6,7].

### General incidence of leukemias

For Mexico City, the frequency of leukemia is higher than those for other cities. For cities in Canada, the USA, or the UK, the SAAIRs are 50.8, 46.9, and 40.8 per million, respectively [21], whereas the SAAIR for Mexico City was 57.6 per million children, as determined in this study. There are several factors that could affect the results: 1) Length of study period. Due to funding constraints, we were able to carry out our study only over a two-year period; therefore, the representativeness of the sampling could be questioned. However, the SAAIR is similar to those published by IMSS for the years 1996-2000 [6] and 1996-2002 [7] (58.4 and 55.4 per million children, respectively). 2) Value used in the denominator. Although we used 2005 data (then the most current official information) for the denominator in the calculations for 2006 and 2007, we are confident that little error was introduced, because over the last 20 years, the population of Mexico City has remained stable [16]. 3) Scope of sampling. This report does not contain information about the children treated in private institutions. However, because in Mexico City, nearly 97.5% of children with leukemia receive medical care in public institutions [12], the exclusion of this small percentage of patients in private institutions should not affect the main conclusions of the current study. In fact, inclusion of said cases would result in a larger numerator, thus leading to an even higher incidence rate. For these reasons, we think that the above-mentioned considerations did not affect the results. We had the opportunity to corroborate only the concordance between the cases of IMSS patients, which were registered in this study, and the data from the MRCC. For the data obtained from the other hospitals, there was no other registry with which to corroborate that the information was complete. Nevertheless, for this study, the registering of cases was done actively, in that a nurse visited the participating hospitals daily in order to identify any child diagnosed with suspected leukemia. Once so diagnosed, the patient was followed until the diagnosis was confirmed. The information in this study was compared to the admissions lists of the hospitals in order to verify that no patient, who had been admitted with the diagnosis of leukemia, had been overlooked by the nurses that performed the data collection. Another factor that gives us confidence in the numerator obtained in this study is that the SAAIR of leukemias (57.6 per million) is similar to those reported in studies by the IMSS (55.4 and 58.4 per million [6,7]). The especially high incidences, reported for children from Mexico City [6] and Costa Rica [5]; for Hispanic children in Florida [22], Los Angeles [23], and Texas [24] in the USA; and for Hispanic children in general as reported by the SEER and the CDC (Table 4) [1-3,25,26], are due to the incidences of lymphoid leukemia in these populations. This finding is very interesting because, according to data from El Salvador [6], Argentina [27], and Brazil [28] and according to the International Report of Cancer in Children, populations in other regions of Latin America do not have higher incidences of lymphoid leukemia than do populations of Caucasian origin [21].

**Table 4:** Comparison of standardized average annual incidence rates of lymphoid leukemias per million children from cancer registries

		Study	Parameters		
		
Area	Source of data	Population	Age range years ye	Period	SAAIR^a^
Mexico City	Present Study	**Mexicans**	**0-14**	**2006-2007**	**49.5**
USA	SEER^b ^[25]	All races	0-14	2007	35.0
	CDC^c ^(NPCR) [26]	All races	0-14	2003-2007	37.0
		**Hispanics**	**0-19**	**2003-2007**	**46.0**
	ACSSR^d ^[2]	**Hispanics**	**0-14**	**2002-2006**	**46.7**
Texas	TCR^e ^[24]	All races	0-14	1999-2008	41.4
California	Wilkinson et al. [1]	**Hispanic**	**0-14**	**1988-1998**	**51.1**
		non-Hispanic White	0-14	1988-1998	40.8
Florida	Wilkinson et al. [1]	**Hispanic**	**0-14**	**1988-1998**	**49.2**
		non-Hispanic White	0-14	1988-1998	37.1
Costa Rica	Monge et al. [5]	**Costa Rican**	**0-14**	**1981-1996**	**43.1**
El Salvador	Mejía-Aranguré et al. [6]	**Salvadoran**	**0-11**	**1996-2000**	**34.2**
Brazil (Sao Paulo)	de Camargo B et al. [28]	**Brazilian**	0-19	**1998-2002**	**47.5**

^a ^SAAIR: standardized average annual incidence rates; ^b ^SEER: Surveillance, Epidemiology and End Results Program in United States of America (nine areas: Atlanta, Detroit, San Francisco, Seattle, Connecticut, Hawaii, Iowa, New Mexico and Utah);^c ^CDC (NPCR): Center for Disease Control, Division of Cancer Prevention and Control, National Program of Cancer Registries in USA; ^d ^ACSSR: American Cancer Society, Surveillance Research (13 SEER cancer registry areas: Atlanta, Detroit, Los Angeles, San Francisco, San Jose Monterey, Seattle, Alaska Native Tumor Registry, Connecticut, Georgia, Hawaii, Iowa, New Mexico, and Utah); ^e ^TCR: Texas Cancer Registry, Texas Department of State Health Services, Cancer Epidemiology and Surveillance Branch.

The high incidence in some Hispanic groups may have resulted from an artifact in the data, or have been due to environmental or genetic factors. In previous reports, there may have been an artifact in the IMSS data, which resulted in an overestimation of the incidence. The following scenario might explain such a situation: unemployed parents, on learning that their child had leukemia, would seek employment that would provide access to medical benefits from IMSS. The child would then be included in the numerator, without being represented in the denominator, thereby leading to an overestimation of the incidence. (A similar situation could be envisioned for the Hispanic population in the USA, many of which are undocumented workers. Illegal aliens may be forced out of hiding to seek medical attention for their child, thereby increasing the numerator). It should be noted that the high incidence demonstrated in the current study agrees with those found in prior reports based on IMSS data, implying that the prior reports were not biased, despite having come from only one institution.

Another possible explanation of the higher incidence among some groups of Hispanics is that exposure to carcinogenic agents is greater among Hispanics in the USA than it is for other ethnic groups [29], as a significant portion of Hispanic immigrants in the USA are employed in high-risk occupations, such as agriculture in which pesticides are used. This is especially true for Florida, California, and Texas [29], the states in which higher incidences of ALL among Hispanic children have been reported [22-25]. We have previously reported that, in Mexico City, both exposure to carcinogenic agents in the workplace and agriculture-related occupations were risk factors for AL in children [30]. However, in this study, for the boroughs of Mexico City, no correlation was found between agricultural hectares and the incidence of ALL. Another possibility is that genetic factors could explain why Costa Ricans, Mexicans, and Hispanics in the USA have the highest incidences of ALL [23]. In contrast, the SAAIR of AML for the study population was 6.8 per million, a value similar to data from Canada, the USA, and the UK (SAAIRs of 6.3, 6.0, 6.3 per million, respectively) [21].

### Incidence of leukemia by age group

First, it should be noted that, when the age groups for ALL are compared across various groups, the incidence for children 1-4 years of age were consistently greater than those for 10-14 year olds: for the majority of Mexican states, two-fold greater (a finding in agreement with the IMSS data); for the state of *Nuevo León*, three-fold; and for developed countries, more than three-fold, even though the rate of ALL is not so high [7]. In the current study, the incidence for 1-4 year olds was 2.6-fold that for the 10-14 year olds. This relation has been associated with socio-economic level and with the possibility that a hypothetical infectious agent may be involved [13]. It is interesting that the frequency of ALL for *Nuevo León*, a Mexican state that borders the USA, is similar to that for the Caucasian population of the USA [7], the fact that *Nuevo León *has the highest MHDI of Mexico, suggests the possibility that, among Hispanic children who enjoy a higher quality of life, the incidence of ALL tends to follow that of developed countries. Further studies are required to validate this hypothesis.

### Immunophenotypes

In this work, the frequency of immunophenotyping was 96.4%, a value higher than those in other published data from Mexico [8] but similar to those in reports from developed countries [31]. The frequency of precursor B-cell ALL was similar to those in other reports from Mexico [8,32,33]; the frequency of T-cell ALL was lower than that (23.6%) in a previous report from IMSS [30], but similar to those in the latest reports from the *Instituto Nacional de Pediatría *[32,33].

### Ratio of high risk to standard risk for ALL

The higher incidence of ALL among children over ten years of age is important because all children of this age are generally considered at high risk for relapse [31,34]. In Mexico City, the ratio of high-risk children to standard-risk children is 1:1, a value in sharp contrast with those for populations attended in hospitals outside Mexico, e.g., St. Jude Children's Research Hospital (Memphis, Tennessee, USA) where the ratio is 1:3. Thus, only 25% of the children at Saint Jude [35] are at high risk, compared to 50% in Mexico City [36].

The importance of a high incidence of ALL at an early age is that it is a predictor of a higher frequency of a genetic rearrangement that has a good prognosis for ALL patients [37]. In Mexico, genetic rearrangements (such as ETV6/RUNX1) that have a good prognosis have been found at a frequency below those of developed countries [38], whereas genetic rearrangements with a bad prognosis have been reported at a higher frequency [38]. Of note is the high frequency found for MLL/AF4 in Mexico City, because this rearrangement, which has a very bad prognosis, has been related with different intrauterine exposures [39].

### AML

For Hispanic populations, AML M3 has been proposed as the predominate subtype [7,40]. This idea was not corroborated in the present work: AML M3, with a frequency of only 10.7%, was not the predominant subtype in the population studied.

### Peak ages for leukemias

In a previous study in Mexico City, peaks of incidence of ALL were at 2-3 and at 6-8 years of age [8]; here, as shown in Figure 1, two peaks were found, but at 1-6 years of age and at 9-10 years of age. In other Hispanic populations in which the incidence of ALL is high [41], a similar situation seems to exist, that of there being two age peaks, one early, one late. A small peak for T-cell ALL occurred at 1-4 years, similar to that published by the IMSS from Mexico [8].

### Incidence of leukemias by borough and correlation with MDHI, number of cultivated hectares, and average number of persons per household

In this study, there was a strong correlation between the incidence of precursor B-cell immunophenotype and the average number of people per household in the boroughs of the city. This finding supports the infectious agent hypothesis, because a child living a crowded household would have a higher risk of being in contact with infectious agents [13]. In our report, the incidence of AML was not correlated with the agriculture hectares, average number of people per household, or MHDI. The incidence in Mexico City is similar to that of other cities in the region [21].

## Conclusions

We conclude that the frequency of AL, especially of ALL, in Mexico City is among the highest in the world, similar to those of Hispanic children populations in the USA or Costa Rica [1,3,5]. Our result showed that this high frequency in Mexico City was not due to any bias that could have existed in prior reports that were based on data from only one institution. The early peak of precursor B-cell ALL occurred at the same ages as those found for developed countries; however, a second peak, at nine years of age and older, was found for children in Mexico City. The frequency of T-cell ALL was similar to those in developed countries; however, here, a peak at an early age was found. It is possible that an infectious agent could be related with the high incidence of ALL in Mexican children. We hypothesize that a lower socio-economic level and infectious agents could be related with the higher incidence of ALL among Hispanic populations.

We think that the establishment of population-based registries in other jurisdictions of Mexico would help track the incidence of these disease--information that may be useful in determining possible causal agents in the environment or other factors that play a role. Such information is also useful to decision-makers in planning for the future needs of the health-care system.

## Competing interests

The authors declare that they have no competing interests.

## Authors' contributions

JMMA and MLPS conceived and designed the study, analyzed the data, and wrote the first draft of manuscript. AFG and RBR designed the study, analyzed the data, and provided guidance to all aspects of this project. JFL, DADR, RAS, MCRZ, RPA, AMA, RRL, LEH, MAdCM, EJH, AMS, EMDA, VBM, JGPG, MMVA, FJAR, JRTN, JDFCH, RCC, PHAG, VCBM, MCOA, and MAI registered, encoded, and analyzed the data. All authors read and approved the final manuscript.

## Pre-publication history

The pre-publication history for this paper can be accessed here:

http://www.biomedcentral.com/1471-2407/11/355/prepub

## Supplementary Material

Additional file 1 **Table S1**. Childhood population in boroughs of Mexico City by age group, according to the intermediate census of 2005. Population by each borough and by age group. Click here for file
