# Supplementary material for: Childhood acute leukemias are frequent in Mexico City: descriptive epidemiology
Source: BMC Cancer. 2011 Aug 17;11:355. doi: 10.1186/1471-2407-11-355 (PMC3171387; doi:10.1186/1471-2407-11-355)
Supplement: Additional file 1 — Table S1. Childhood population in boroughs of Mexico City by age group, according to the intermediate census of 2005. Population by each borough and by age group. [file 1471-2407-11-355-S1.DOC]

**Additional file 1, Table S1.**

**Childhood population in boroughs of Mexico City by age group, according to the intermediate census of 2005***

| **Boroughs of** |  | **Age** | **Group** |  |  |
| --- | --- | --- | --- | --- | --- |
| **Mexico City** | **< 1 year** | **1-4 years** | **5-9 years** | **10-14 years** | **Total** |
| Álvaro Obregón | 10,289 | 45,903 | 55,779 | 58,195 | 170,166 |
| Azcapotzalco | 5,355 | 23,797 | 29,501 | 32,221 | 90,874 |
| Benito Juárez | 3,231 | 13,497 | 16,985 | 18,256 | 51,969 |
| Coyoacán | 7,183 | 32,237 | 41,085 | 43,767 | 124,272 |
| Cuajimalpa de Morelos | 2,965 | 12,863 | 15,647 | 15,374 | 46,849 |
| Cuauhtémoc | 6,122 | 26,599 | 32,489 | 35,285 | 100,495 |
| Gustavo A. Madero | 16,298 | 73,385 | 91,418 | 97,181 | 278,282 |
| Iztacalco | 5,387 | 22,974 | 28,860 | 31,095 | 88,316 |
| Iztapalapa | 29,755 | 129,462 | 160,080 | 164,524 | 483,821 |
| La Magdalena Contreras | 3,630 | 15,542 | 19,452 | 20,111 | 58,735 |
| Miguel Hidalgo | 4,066 | 17,215 | 21,461 | 22,157 | 64,899 |
| Milpa Alta | 2,166 | 9,364 | 11,498 | 12,221 | 35,249 |
| Tláhuac | 6,131 | 26,248 | 32,903 | 34,198 | 99,480 |
| Tlalpan | 8,660 | 39,080 | 47,824 | 49,618 | 145,182 |
| Venustiano Carranza | 5,682 | 24,821 | 32,184 | 34,445 | 97,132 |
| Xochimilco | 6,428 | 27,757 | 34,413 | 36,302 | 104,900 |
| **Total** | **123,348** | **540,744** | **671,579** | **704,950** | **2,040,621** |

*[16]
